# Relative risks for digestive and respiratory episodes in patients with HIV co-infection

**DOI:** 10.1186/1471-2334-14-S4-P45

**Published:** 2014-05-29

**Authors:** Mirandolina Prişcă, Dana Negru, Laura Nicolescu, Teodora Olaru

**Affiliations:** 1 Arad County Emergency Hospital, Arad, Romania; 2 Public Health Department, Arad, Romania

## 

HBV, CMV or TB co-infection worsen progression of HIV, due to specific pathogenicity of etiologic agents and antimicrobial therapy which is required to be initiated.

Authors have tried to establish a link between HBV, CMV or TB co-infection and the risks for emergence of digestive and respiratory episodes. We have used data from the medical records of HIV-AIDS department of Arad County Emergency Hospital for HIV patients in evidence in 2013. Data were analyzed SPSS.14.0 for Windows, MedCalc and Epi Info Analysis.

Of the 149 patients 38 presented HBV, CMV or TB co-infection (25.5%) and statistically was significant in the history of their recorded adverse reactions that changing ART regimens was required (P=0.0324), they also displayed digestive and respiratory episodes P=0.0057 and P=0.0033, being at relative risk of 1.7344 to 1.6692 respectively for these episodes compared with HIV patients co-infection.

HIV patients with co-infection stand at increased risk for digestive and respiratory intercurrent infectious episodes, monitoring them being a basic element for their quality of life and for survival rates improvements.

